# Leave It to Large Language Models! Correction and Planning with Memory Integration

**DOI:** 10.34133/cbsystems.0087

**Published:** 2024-03-27

**Authors:** Yuan Zhang, Chao Wang, Juntong Qi, Yan Peng

**Affiliations:** ^1^ School of Future Technology, Shanghai University, Shanghai, China.; ^2^ Institute of Artificial Intelligence, Shanghai University, Shanghai, China.

## Abstract

As humans, we can naturally break down a task into individual steps in our daily lives and we are able to provide feedback or dynamically adjust the plan when encountering obstacles. Similarly, our aim is to facilitate agents in comprehending and carrying out natural language instructions in a more efficient and cost-effective manner. For example, in Vision–Language Navigation (VLN) tasks, the agent needs to understand instructions such as “go to the table by the fridge”. This understanding allows the agent to navigate to the table and infer that the destination is likely to be in the kitchen. The traditional VLN approach mainly involves training models using a large number of labeled datasets for task planning in unseen environments. However, manual labeling incurs a high cost for this approach. Considering that large language models (LLMs) already possess extensive commonsense knowledge during pre-training, some researchers have started using LLMs as decision modules in embodied tasks, although this approach shows the LLMs’ reasoning ability to plan a logical sequence of subtasks based on global information. However, executing subtasks often encounters issues, such as obstacles that hinder progress and alterations in the state of the target object. Even one mistake can cause the subsequent tasks to fail, which makes it challenging to complete the instructions through a single plan. Therefore, we propose a new approach—C (Correction) and P (Planning) with M (Memory) I (Integration)—that centered on an LLM for embodied tasks. In more detail, the auxiliary modules of the CPMI facilitate dynamic planning by the LLM-centric planner. These modules provide the agent with memory and generalized experience mechanisms to fully utilize the LLM capabilities, allowing it to improve its performance during execution. Finally, the experimental results on public datasets demonstrate that we achieve the best performance in the few-shot scenario, improving the efficiency of the successive task while increasing the success rate.

## Introduction

In embodied tasks, the agent needs to comprehend the instructions in natural language to execute the pertinent operations [1,2]. For instance, Vision–Language Navigation (VLN) is a widespread embodied task with the primary objective of comprehending natural language instructions, followed by devising suitable routes and operations using data from numerous sensors and executing them accordingly. Previous studies of embodied tasks focus on comprehension of instructions and decomposition into subtasks within the embodied tasks [3,4]. Recent studies have revealed an impressive finding about LLMs, in that they can not only handle traditional word processing tasks but also exhibit a degree of reasoning ability to tackle diverse issues by appropriate prompts [5–10]. This may be due to the presence of abundant internalized knowledge about the world in LLMs’ pre-training process [11–16]. Typically, traditional VLN tasks incorporate a specific model to comprehend instructions and plans. However, due to the development of LLMs’ reasoning capability, some researchers have attempted to use LLMs as planners in embodied tasks, but usually with unsatisfactory results. This leads to a pressing question: How can we maximize the reasoning power of the LLMs and the commonsense knowledge within them to improve planning in embodied tasks?

Previous research has mainly utilized lexical analysis or trained models to decompose instructions on embodied tasks [17–19]. This approach needs significant human resources to create expert data for training. With the advent of LLMs, some studies have recently integrated LLMs with embodied tasks and used LLMs as the core planner [20–22]. Additionally, some research has been devoted to the development of generic embodied intelligence that relies on language models [23]. However, most current research uses overall planning [3,24,25] to obtain reasonable global subtask sequences. Although the generated plan may be logically complete, the agent will encounter many obstacles during the actual process of execution. Relying only on the previous global plan may cause the agent to fail. For example, in the case of a series of subtasks starting with *Pick up an egg*, failing to find the *egg* at the beginning would result in the outright failure of the entire plan. We expect to use the power of the language model to adjust the dynamic plan, such as further attempts to find the *egg* in the fridge. Meanwhile, we anticipate that the agent should possess memory and abstraction capacities in successive tasks to accumulate experience in upcoming tasks akin to human beings. For instance, in the previous example, once the *egg* is located inside the fridge, the agent should learn to open the fridge and locate the item during exploration. Without memory, the agent will need to perform basic exploration during execution repeatedly. Experience comprises high-level information abstracted from memory, without which the agent will make similar errors in tasks. In practical scenarios, tasks tend to be adaptable and subject to change. Our framework thus requires extensibility to accommodate various embodied tasks.

However, expanding or replacing LLMs can introduce derivative issues, such as hallucination [26,27], which will result in unstable output. From this, it is necessary to ensure the framework’s robustness fully. Therefore, a superior framework should address the following challenges: Challenge 1: Identifying a dynamic and efficient method to utilize the capabilities of LLMs for embodied tasks fully. Challenge 2: Establishing mechanisms for memory and abstract experience that enable more efficient planning based on information from memory while using abstracted experience in successive tasks to circumvent faulty planning. Challenge 3: Ensure the entire framework is scalable and robust, and allow it to be used as a lightweight framework without additional training costs.

To tackle these concerns, we proposed the C (Correction) and P (Planning) with M (Memory) I (Integration) framework, which effectively utilizes the reasoning abilities of LLMs through collaborative efforts among multiple ancillary modules. This will allow for sturdy and versatile task planning. Its objective is to enable executing embodied tasks at the least demanding level, and planning performance will correspondingly improve as the language model develops and reasoning capability improves. Specifically, for Challenge 1: We use the combination of the Memory Module and the Planner Module in CPMI. This allows the language model to fully use commonsense knowledge in planning and demonstrate the ability to memorize and summarize experience in successive tasks. For example, in practice, we may encounter commands whose purpose is unclear, such as *I am thirsty*. We can now understand the intention underlying the commands through the commonsense knowledge of the language models. In this case, the language model will generate a reasonable subtask: *get a glass of water*, while the Memory Module collects the action and visual information during the execution. As for Challenge 2: upon completing the assigned task or after a dynamic correction, the experience will be summarized from memory to aid in future planning and execution. If we view the agent as the physical body parts of CPMI, such as the arms, feet, and senses, then the LLMs can be seen as the brain. Its responsibilities extend beyond just understanding instructions and providing subtasks, as it also gathers information from the senses to make instant modifications. For Challenge 3: we suggest a module specifically designed to identify and rectify the language model’s output. This will ensure that the accurate sequence of subtasks is produced across diverse language models and task configurations and guarantee the robustness of the framework. Meanwhile, the framework’s modules can be effortlessly expanded via interfaces to facilitate language model development. For instance, more visual data can be integrated into the Memory Module.

Finally, we evaluate the performance of CPMI in the ALFRED [10] simulation environment via comparison and continuous task experiments. The results indicate that our approach achieved superior performance in few-shot situations. We enabled the agent to memorize and abstract experience through the Memory Module’s role, leading to better performance than the standard agent in continuous tasks. To summarize, our key contributions are the following:

• We propose CPMI, a lightweight embodied task framework that enables agents to be embedded under LLMs. Its performance improvements are directly achieved through these models’ development.

• Propose a Memory Module that allows the agent to exhibit memory and higher levels of abstraction by accessing information from the execution process, as well as correcting and refining the trajectory of the execution.

• Through several experiments, CPMI has been shown to achieve state-of-the-art (SOTA) performance on the ALFRED dataset in few-shot scenarios. Moreover, incremental performance gains in successive tasks indicate the benefits of memory and abstraction capabilities.

## Related Work

### Vision–language navigation

Since the Vision–Language Navigation task was first introduced in the room-to-room task, it has garnered widespread attention. In the initial research [10,28,29], people tried to use the end-to-end models to accomplish this task, but the effect was minimal. With the development, people started to explore the modular [30–32] or hierarchical [33–37] solution: to separate the navigation perception task from the decision-making task, most methods have specific information processing modules. The perceived information is then fused to the decision-making module for planning through various ways, so the problem was gradually transformed into a high-level discrete task planning problem and a low-level continuous motion planning problem.

### LLM for VLN tasks

With the increasing interest in large language models (LLMs), numerous studies have attempted to utilize LLMs for exploring novel developmental pathways. For instance, in the VLN task, the agent must navigate in an unseen environment based on the linguistic inputs. Pre-trained LLMs allow us to eliminate expensive data and try to accomplish embodied tasks such as VLN at a lightweight cost. As previously noted, researchers have started experimenting with LLMs for advanced discrete task planning. The work focuses on using pre-trained LLMs to decompose natural language commands into sets of subtasks that agents can execute [21,38–40]. Still, none of these approaches are robust enough. If any obstacles arise while executing subtasks, it could lead to the failure of the entire plan. For example, the overall plan is: *pick an apple, move to the sink, put the apple in the sink*. When the apple is located inside the fridge and cannot be found directly by exploring, the whole plan will fail instantly, instead of replanning based on the commonsense knowledge and changing the plan to: *open the fridge, find the apple*. Therefore, some current work on doing extensions around LLMs [41,42] made us consider: Is it possible to add modules as an adjunct to a large language model to fully utilize its reasoning capabilities and enable the language model to cope with uncertainty in its execution dynamically?

In this paper, we propose a framework that is easy to extend: using the modularity concept, the LLMs are used as the core decision-making part, and an interface is used to access the parts responsible for continuous motion planning, where the top-level LLMs and the low-level Planner are interchangeable. Simultaneously, the rest of the component’s rules can be readily expanded to obtain additional information for the LLMs.

## Methods

This section begins with problem and goal modeling before introducing our CPMI framework. Then, we provide a detailed description of how the LLMs function as the “brain” in our framework.

Our setup includes an agent with visual perception capabilities as the execution unit. This agent can perform actions from the dynamically adjustable action library Π based on the task goal given in high-level natural language instruction *τ*. The agent will attempt to comprehend and fulfill the task requirements step-by-step, utilizing the LLMs as the core for decomposition, planning, and modification without any supplementary training. The following subsections will detail each part’s functions and operation flow.

### Problem statement

As per current research, LLMs are trained on vast data and thus have a significant stock of commonsense knowledge and demonstrate certain reasoning abilities [4,6,43,44]. Hence, we aim to utilize this reasoning capability and commonsense knowledge as the foundation of our planning methodology. Unlike previous research that focused on single tasks, we look toward real-world missions where we should perform a sequential task with multiple instructions. Then, we present the composition of our framework and how it flexibly exploits the capabilities of LLMs to solve successive planning problems in embodied tasks.

CPMI comprises 4 principal modules—Memory Module, Planner Module, Correct Module, and Replan Module—and a Low-level Planner Interface. In detail, the core part of CPMI comprises the Planner Module and Memory Module, where the Planner Module generates an initial subtask sequence by breaking down the given instructions, while the Memory Module serves as the agent’s memory function, allowing access to stored memory content during execution and generating experience based on it. Correct Module ensures the framework’s robustness under multitasking and different language models. Replan Module plays a key role in dynamic planning, allowing for timely adjustments in case of obstacles during task execution. The Low-level Planner interface offers an agent execution operations interface and object extractors, which can be embodied in various Low-level Planners based on the task requirements.

**Fig. 1.**
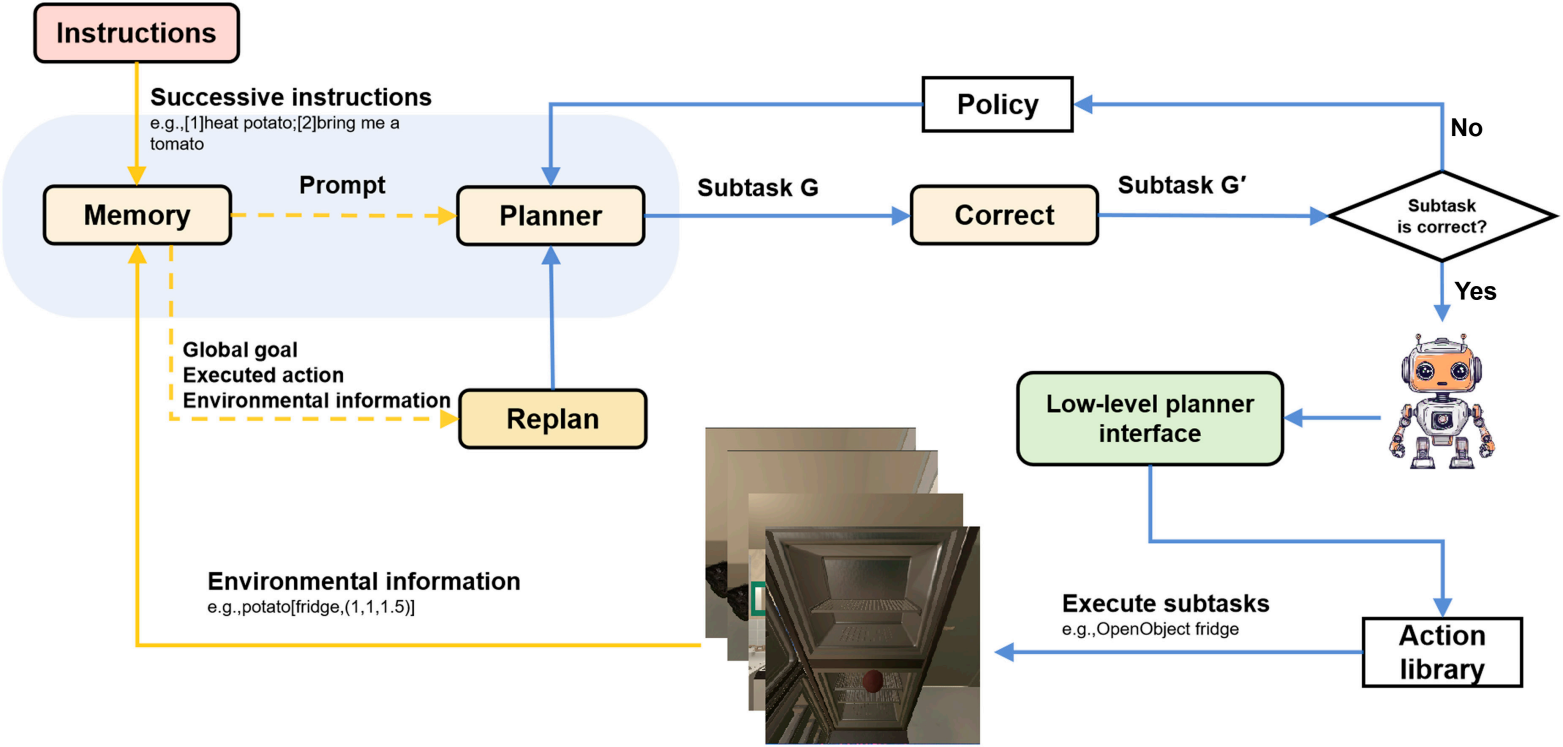
Flowchart of the CPMI. The solid yellow line indicates memory storage, and the dashed yellow line indicates memory output. The brain’s main part comprises the Memory Module and the Planner Module.

The planning process is illustrated in Fig. 1. First, we take the received natural language commands as input to the framework and initiate the process by storing the commands and their sequence number in the Memory Module. Furthermore, we contact the Low-level Planner interface and store the objects obtained from the object extractors in the Memory Module. Lastly, we input the command and the objects into the Planner Module to generate the subtask sequence

$$G=\left(M\cdot g,N\cdot h\right),$$ (1)

where *h* stands for subtasks whose format does not conform to the interface specification, and *g* is a subtask that the agent can directly execute after calling the Low-level Planner interface, such as *Move to cup* and *Pick up cup*. *M* represents the number of subtask *g* while *N* represents the number of subtask *h*. However, the output of the framework can have errors in meaning or structure when using a different language model or performing successive tasks. Therefore, in addition to *G*, it may contain *N* variables *h* that cannot be executed directly. To ensure the framework’s robustness, a module is developed that detects the sequence *G* based on a specific strategy. If any errors are detected, then the Correct Module will make the correction and output the sequence

$$G^{\prime }=\left\{g_{1},g_{2},g_{3}\cdots g_{n}\right\},$$ (2)

At this point, the initialization of the framework is complete, and *G*′ contains only correctly formatted atomic operations *g_n_*. The Memory Module stores the sequence of subtasks *G*′, detected objects, and multiple raw instructions. *G*′ is subsequently passed into the low-level planning interface for execution.

In contrast to the previous singular planning, there is a high likelihood of failure when executing subtasks due to potential obstacles. Therefore, continuous feedback is designed in the execution process and replanning based on information from the Memory Module when a subtask fails. During the execution process, the agent periodically returns a list of objects *O*, consisting of the name, location, and type of the items. The execution trajectory before and after replanning will be stored in the Memory Module. In response to the trajectory correction process, LLMs will summarize the trajectory, which will be used to plan the rest of the instructions.

### Memory module

The fundamental purpose of the Memory Module is analogous to the human recall process and assists the LLM for long-term planning [45]. The agent’s initial identification of objects takes place in an unknown environment through the visual input it receives. Prior research has focused on linking task guidelines with current visual data to emphasize important objects [46]. However, back to the most essential when humans act according to instructions, they do not only rely on their senses to discover the information in the scene but also abstract the relevant information of the task through memory: The apple may not be around when they need to reach for an apple. However, they can recall seeing it by the sink through memory and executing tasks such as *move to sink* and *pick up apple* without random exploration. Meanwhile, individuals tasked with similar activities, such as *retrieving a grape from the kitchen*, should be able to glance at the sink again when passing by, based on prior experiences.

**Fig. 2.**
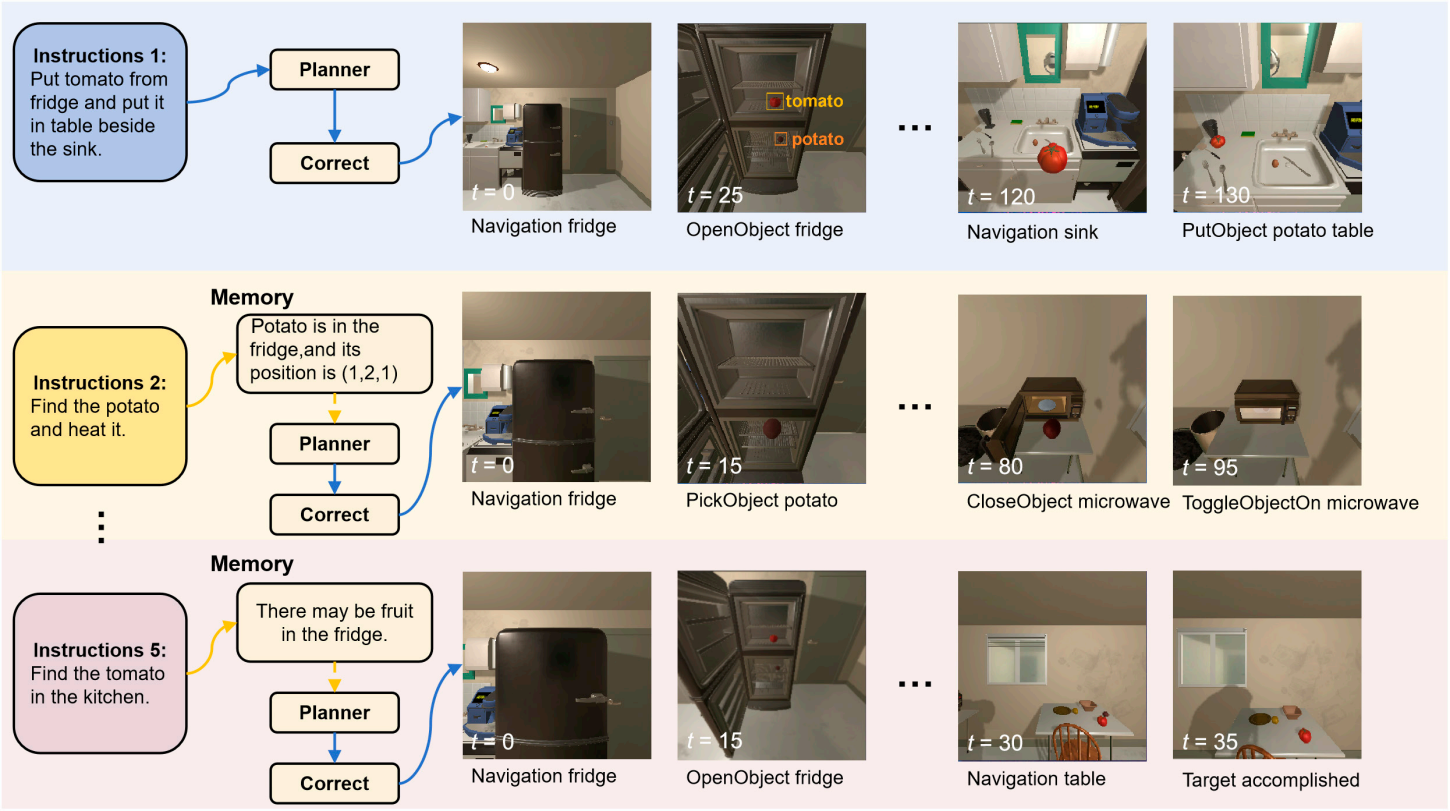
Performance in successive tasks. While carrying out instruction 2, it remembered encountering “potato” in instruction 1. Then, it skipped the search step and went directly to the fridge. In addition, based on the stored experience, the agent opens the fridge to look for the tomato when performing the fifth task: find tomato, because the fridge is in the field of view and the experience informs that there may be food in the fridge.

A Memory Module is established to store crucial task information for this inspiration. This module assists the decision-making process by providing relevant access to the stored information. Meanwhile, the execution trajectory data are also saved for experience abstract. The specific experience abstract process will be presented in the Replan Module (see the “Replan module” section). More specifically, objects’ location, type, state, and interaction type are stored in a tree structure within the Memory Module. The data in the Memory Module can be used as coarse-grained information, while the object extractor in the Low-level Planner can provide fine-grained information. As shown in Fig. 2, the memory mechanism allows the agent to perform successive tasks more efficiently.

Additionally, it is worth noticing that much additional information can be used to increase the success rate, such as action-execution sequences, room symbols for multi-room navigation, etc. Therefore, our CPMI can be easily extended and adapted to different tasks.

### Prompt design

**Fig. 3.**
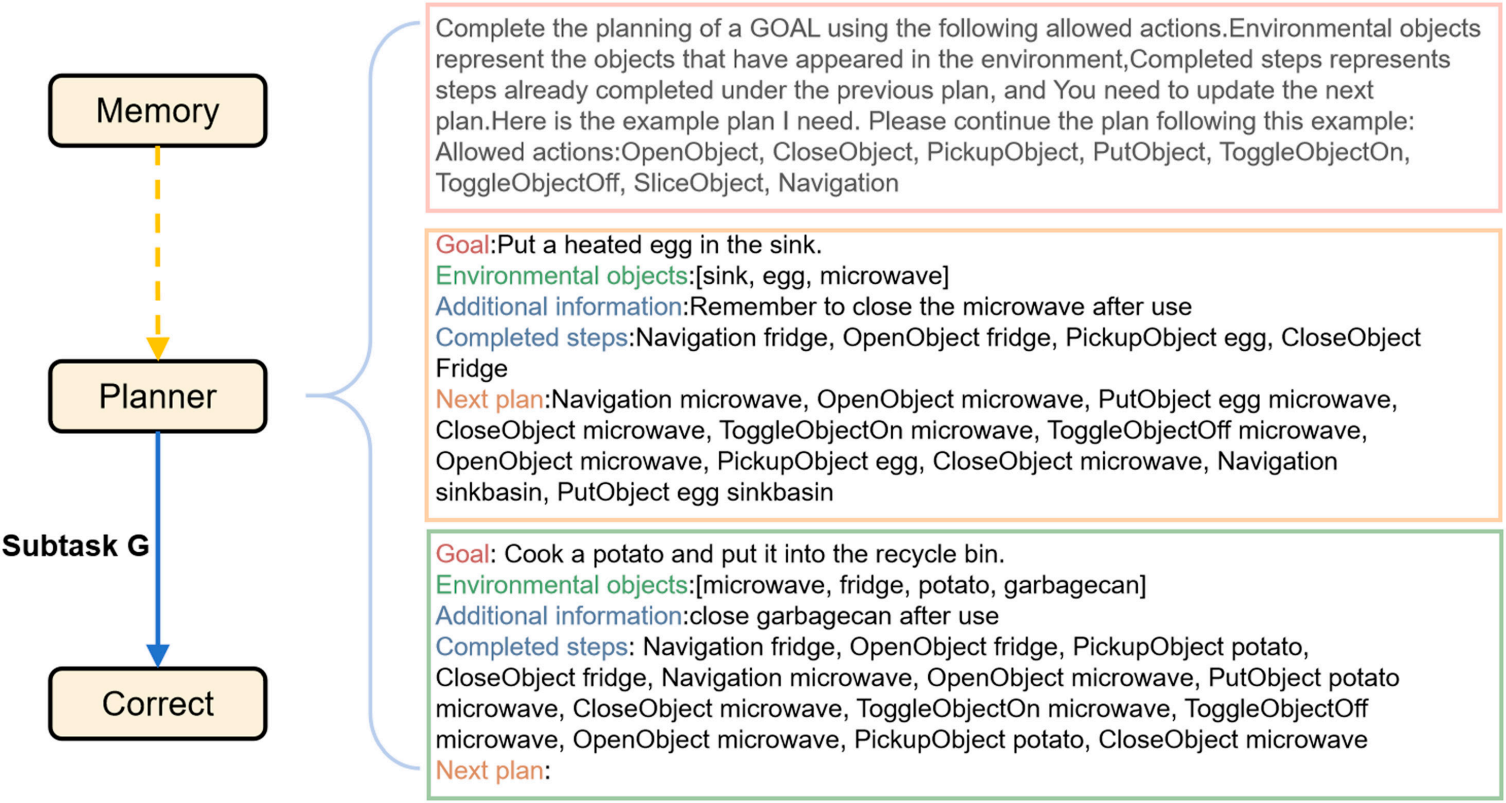
The specific structure of the prompt. Goal represents our final destination, and both Environmental objects and Completed steps consist of information extracted from memory. Additional information comes from the experience gained through historical trajectories.

Typically, traditional embodied task architectures employ a decision model trained on specific data, but this requires a large amount of human data to supervise the training process. Recent research has demonstrated that LLMs can replace traditional models due to the large amount of human behavioral data encoded in LLM training. This led to the derivation of several prompt-based authoring systems [41,42,47–49] where the model can generate plausible behaviors by providing a piece of in-context-contextual cueing [6,50]. Therefore, we expand on the work of [51] and explore different techniques to amalgamate more data for augmenting the quantity of information gathered from the agent’s performance. The final outcome is illustrated in Fig. 3.

Based on prior research, we propose incorporating an Additional Information section dedicated to storing experience summarized by the trajectory stored in the Memory Module. This experience can guide the planning of long-term tasks such as *Use microwave when heating is required*. The precise process of experience generation will be further discussed in the “Replan module” section. In addition, to improve the accuracy of the model’s planning, we use a simple classifier to filter the objects in memory during each planning stage and place priority on objects in the field of view or objects related to the task. For example, the instruction is *heat the soup*. If we observe bowls and knives, we will prioritize the bowls as targets.

### Correct module

**Fig. 4.**
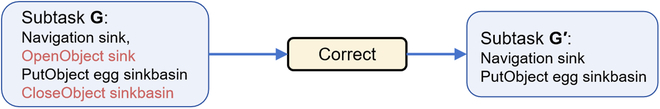
The process of correcting the state of an object. By using the Correct Module, erroneous operations can be detected and removed from subtasks.

Following the initialization process, the Planner Module generates a sequence of subtasks *G*. However, the output of LLMs might be unstable for complex reasons such as hallucination [26,27]. In order to ensure the robustness of the framework, we add the Correct Module to ensure that output is correctly formatted, and the policy of this module can be adaptively changed to match different low-level navigation interfaces or LLMs. Based on the action space of AI2-THOR [52] and the API document, we employ the following strategies:

Correct state of objects: AI2-THOR objects have different state attributes like “*Pickupabl*”, “*Moveabl*”, “*Toggleabl*”, etc. The previous planner’s initial subsequence *G* may contain incorrect operations. For example, *OpenObjectsink* is given in Fig. 4, but the sink is not an open-able object. Then we will determine that the subtask planning is incorrect and add Additional information: sink is not open-able, then return to the Planner Module.

**Fig. 5.**
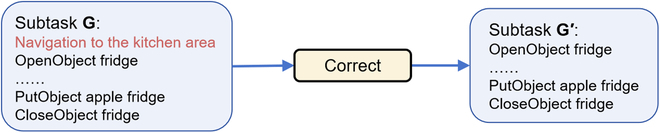
The process of correcting output semantics. If an output is not a limited action, then it should be removed automatically.

Correct semantics of output plans: As our setup is founded on a long-term planning premise, the prompts entered into the LLMs will expand due to objects or additional information as the runtime increases. Occasionally, after our robustness tests, we produce outputs that breach the limit as depicted in Fig. 5. In such cases, we consider it an unenforceable planning error.

### Replan module

After implementing the Correct Module, a logical sequence of subtasks *G*′  = {*g*_1_, *g*_2_, *g*_3_……*g_n_*} is obtained; *g_n_* is in the format provided by the Low-level Planner, such as *g*_1_: *PickUpObject apple*. However, when the brain cannot get any feedback on the action execution, each decision is like a big gamble. Therefore, various combinations are tested to integrate environmental information into the Agent fully. With the aid of feedback and the replanning mechanism, bridges can be established between the arms, legs, and brain. Through this bridge, the model will be able to get feedback from the execution of the action and make timely modifications, which importantly enhances the success rate of planning tasks.

**Fig. 6.**
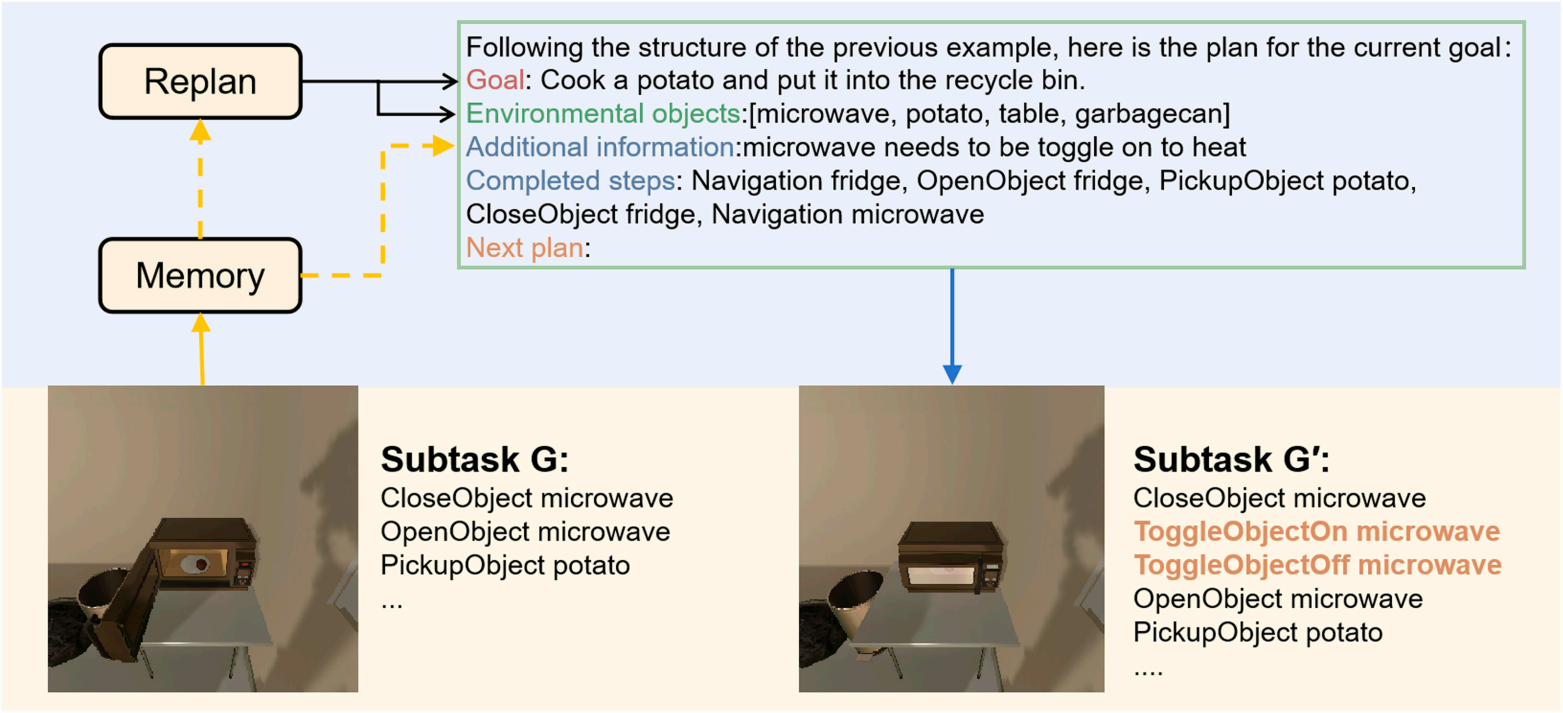
The diagram shows the process of replanning. The yellow solid line represents memory storage, whereas the dotted line represents memory extraction, and the experience previously stored in the Memory Module will guide this replanning.

Specifically, various historical information is stored in the Memory Module during the execution process, including the observed objects, executed actions, and current goals. Suppose we identify a failure in subtask execution (like being stuck in an endless loop) or the subtask remains incomplete after a certain step. In that case, we determine that the subtask cannot be executed. After that, we will extract the global list of objects from the Memory Module, and we will extract the global list of objects from the Memory Module, particularly those present in the current view or relevant to the subtasks. Meanwhile, if there is experience left over from previous tasks in the Memory Module, we will use it as the content of Additional Information, which will eventually be updated based on the planner’s prompt to get the new plan from the model. For instance, based on prior experience, we learned that the *microwave needs to be toggled on to heat*, as seen in Fig. 6. Therefore, we include this experience to ensure proper guidance for the rest of the planning process.

To be more precise, experience is derived from failure. The Memory Module provides the agent with a complete execution trajectory, labeled every time replanning is performed. The aim is to obtain the trajectory that has been labeled and to summarize the task with a prompt and 2 in-context-examples while storing the gained experience in the Memory Module. Simultaneously, if the modified trajectory successfully completes the task, it is stored as a memory in the Memory Module.

## Results 

The experiments begin with a concise overview of the environment setup. Then, we compare CPMI with several baselines in the simulation environment. We mainly verify how the embodied agent using LLMs for planning performs in the unseen environment. Specifically, we need to answer the following questions:

• Can CPMI effectively perform the task in a few-shot scenario?

• Is it possible to use the Memory Module for successive tasks to improve subsequent task speed?

### Experiment detail

Dataset: To evaluate our approach’s performance, we require a simulation environment to model realistic navigation interaction tasks. Several benchmarks are available to test agents’ ability to follow natural language instructions, and ALFRED [10] is currently the most popular choice. The ALFRED dataset provides a visual demonstration in a single environment, where step-by-step natural language descriptions of high-level goals and low-level instructions are provided for each task. On average, agents are given natural language instructions to complete more than 70 steps of a long-running task. Notably, the ALFRED benchmark builds on the AI2-THOR simulator [52]. Thus, the main tasks in it are focused on the kitchen scenario, which contains explicitly tests seen for 1,533 episodes, tests unseen for 1,529 episodes, valid seen for 820 episodes, and valid unseen sets of 821 episodes, which includes different types of operation tasks such as *pick*_*heat*_*then*_*place*, *pick*_*cool*_*then*_*place*, etc.

Metric: In this paper, we first evaluate the method using the official ALFRED metrics, which include success rate (SR) and goal-conditioned success rate (GC) in both seen and unseen environments, where SR evaluates whether all subtasks have been completed, and GC is used to measure the proportion of subtasks completed in an episode. There are also path-weighted metrics to rationally assess the efficiency of completing tasks.

Baseline: As of publication, the SOTA method on ALFRED’s leaderboard is prompter [25], which uses LLMs to replace FILM’s [3] semantic search module and produces a replacement for Streamline by prompting on the model. Meanwhile, we conducted comparative experiments using the top-ranked open-source models on the leaderboard to compare performance in the few-shot case.

Experiment Setup: To validate our approach, we sample 100 task examples evenly from the ALFRED dataset, categorized by task types as part of in-context learning in the Planner Module. We then retrained the model for instruction decomposition to compare its few-shot ability against other methods while leaving the original parameters unchanged for all other parts.

In the comparative experiments, we extend the research of Song et al. [51]. We solely employ the K-Nearest Neighbor retriever to select the most pertinent example from 100 task instances. However, taking advantage of memory mechanisms, we will gradually extend the search scope to trajectories stored in the retrieval Memory Module. As for the Low-level Planner, the navigation and observation interfaces provided in Hierarchical Language-conditioned Spatial Model (HLSM) [24] are selected, and the subgoal model is retrained to perform the comparison under a few shots. The subgoal model undergoes few-shot comparison training utilizing 100 datasets, which are also deployed to train the instruction decomposition modules of the open-source methods used for evaluation. Unless expressly stated otherwise, the default selection for large-scale language models in the Planner Module is text-davinci-003.

To examine the role of the mnemonic mechanism in our approach, we experimented using a successive task. We utilized scenario files from the ALFRED dataset to generate 3 consecutive instructions based on the task type and associated objects. These instructions are evaluated separately for completion metrics.

### Results analysis

The results of the comparison experiment: We conduct an experiment comparing the high-performing models on the ALFRED dataset with our approach. As demonstrated in Table 1, we arrive at the following conclusions:

**Table 1.** Results of the comparison experiment, where bold highlights the optimal performance for the current setting. CPMI is optimal for most metrics in few-shot situations and exceeds the original performance when combined with HLSM’s low-level planning interface.

Method	Test seen				Test unseen			
	SR	GC	PLWSR	PLWGC	SR	GC	PLWSR	PLWGC
*Full data setting*								
Step-by-step instructions								
FILM	28.83	39.55	11.27	15.59	27.8	38.52	11.32	15.13
LWIT	30.92	40.53	**25.90**	**36.76**	9.42	20.91	5.60	16.34
LGS-RPA	40.05	48.66	21.28	28.97	35.41	45.24	15.68	22.76
Prompter	**53.23**	**63.43**	25.81	30.72	**45.72**	**58.76**	**20.76**	**26.22**
High-level goal instructions only								
HLSM	25.11	35.79	6.69	11.53	16.29	27.24	4.34	8.45
FILM	25.77	36.15	10.39	14.17	24.46	34.75	9.67	13.13
LGS-RPA	33.01	41.71	16.65	24.49	27.80	38.55	12.92	20.01
Prompter	**49.38**	**55.90**	**23.47**	**29.06**	**42.64**	**59.55**	**19.49**	**25.00**
*Few-shot setting*								
Step-by-step instructions								
HLSM	0.56	5.71	0.32	4.80	0.46	3.24	0.35	2.73
FILM	0.00	3.31	0.00	2.42	0.20	6.68	0.07	4.13
High-level goal instructions only								
LLM-Planner + HLSM	18.20	**26.77**	-	-	16.42	23.37	-	-
CPMI + HLSM	**18.56**	26.15	**5.53**	**8.65**	**17.79**	**24.42**	**3.76**	**7.58**

1. It is apparent that the CPMI method outperforms all other methods in most cases in the few-shot scenario, indicating that subtask planning can be achieved more effectively through our approach.

2. Notably, only a tiny amount of current data is required for the prompt to achieve similar performance levels to a model trained with an entire dataset, improving unseen SR metrics substantially. When fused with the Low-level Module of HLSM, the SR metrics of our method in the unseen environment surpass those of HLSM.

3. We find that our method achieves similar performance in both seen and unseen environments, due to the Memory Module storing successful subtask execution trajectories and the agent having access to a more refined in-context-learning example during execution. However, it is evident that contemporary pre-training methods for modeling have yielded a 45.72% success rate in an unseen environment, and we are still far from their performance. Our PLWSR metric is lower than most methods. This is due to the fact that the current language model acquires less information than the traditional method variant that utilizes the attention mechanism, resulting in some redundant operations during replanning.

The results of the successive instruction experiment: Initially, we eliminate the memory mechanism and experience summary function in the CPMI, and the Memory Module is solely employed to save all the seen objects and instructions. Table 2 presents the results of our successive instruction experiments. The following are apparent from the experimental results:

**Table 2.** Results of models with initial memory under long-term tasks. The CPMI with the memory reflection feature provides a significant improvement in the subsequent instruction metrics, especially the PLWSR, but the overall SR improvement seems slightly insufficient, as the limiting success rate comes more from the low-level action execution.

Methods	SR	GC	PLWSR	PLWGC
Instruction 1				
CPMI (with memory)	17.52	23.47	3.60	7.38
CPMI	13.42	19.71	2.27	4.13
Instruction 2				
CPMI (with memory)	18.77	25.53	4.43	8.13
CPMI	13.21	19.81	2.01	3.76
Instruction 3		
CPMI (with memory)	18.91	25.60	5.65	9.29
CPMI	13.41	19.79	2.39	4.16

**Table 3.** Ablation of CPMI’s components

Methods	Memory	Replan	Correct	SR	GC
CPMI (without Memory)	✗	✓	✓	13.21	18.87
CPMI (without Replan)	✓	✗	✓	4.65	11.24
CPMI (without Correct)	✓	✓	✗	7.78	19.98

1. CPMI with memories had a higher performance during the execution of the first instruction. Still, the performance differences observed in these conditions came mainly from filtering the objects according to the task instructions.

2. The findings of instructions 2 and 3 indicate that the CPMI’s performance is markedly enhanced by experience when utilizing the Memory Module. This is particularly evident in metrics that are linked to the average step size. Conversely, using the CPMI without the memory function does not yield any change in performance. These results suggest that experience leads to greater efficiency by avoiding superfluous operations. However, our findings indicate that there is no notable increase in success rate when multiple instructions are given. We speculate that while recalling may enable more efficient navigation and subtask decomposition, subtask failure typically stems from the unattainable position of the goal object or the unreachable object, both of which are not resolved solely by memory.

**Fig. 7.**
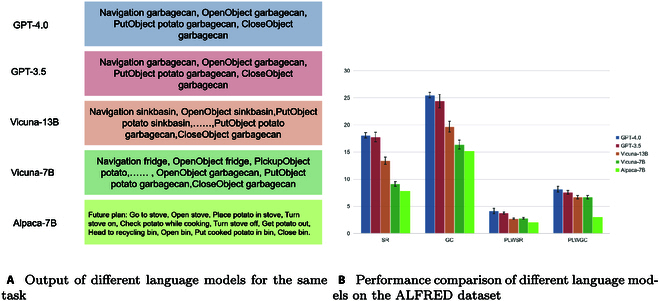
Comparison of performance after using different language models as the core of planning. (A) Output of different language models for the same task. (B) Performance comparison of different language models on the ALFRED dataset.

The result drawn by using different LLMs: Ultimately, to ascertain the robustness of the CPMI framework, we chose different open-source language models as the planning kernel to compare the benefits of the improved performance of the language models. The findings in Fig. 7 indicate that varying language models result in significant differences in planning, with larger models exhibiting enhanced reasoning capabilities. Specifically, Fig. 7 Bon the right indicates that employing GPT-4.0 technology as the planning core surpasses the other models in a range of metrics. It is closely trailed by GPT-3.5. However, as the model scale increases, the trend in the success rate (SR) growth gradually diminishes. We speculate that this phenomenon may be attributed to the fact that the ultimate causes of failure lie in the inability to execute subtasks using low-level interfaces, rather than failures in the planning phase. Additionally, we observed that larger model specifications tend to yield better performance. For instance, in the case of GPT-3.5 and GPT-4.0, it is likely due to the more diverse data used in training GPT-4, which contributes to enhanced context understanding. Notably, the Correct Module’s robustness guarantee allows for a practical subtask list with minimal modifications.

The left part of Fig. 7A displays the specific outputs employing various language models as planning cores, all utilizing the example from Fig. 3 as the target. The results demonstrate that GPT-4.0 and GPT-3.5 produce satisfactory outputs, while smaller language models frequently struggle to comprehend contextual examples and task requirements, resulting in incoherent or improperly structured subtask lists.

### Ablation studies

To establish the role of each module in CPMI, we conducted an ablation experiment where we selectively removed the Replan Module, Memory Module, and Correct Module in CPMI in several batches. We subsequently assessed the performance of these iterations through the ALFRED dataset, resulting in the following outcomes: Firstly, it is evident that the ablation of the Replan Module results in a prominent SR drop. As we suspected, direct attainment of the objective is difficult in the absence of dynamic plan modifications. In a similar vein, the Correct Module’s absence exposes the uncertainty of the comprehensive language model pertaining to complex tasks and lengthy contextual information, resulting in a substantial drop in SR and GC. Finally, it is evident from the ablation results of the Memory Module that only a minor part of the effectiveness can be enhanced by the memory mechanism in single instructional tasks. However, when combined with Table 2, it becomes apparent that the memory module’s improvement is more remarkable under successive directions.

## Discussion

In this work, we present the CPMI framework, which achieves memory and experience abstract functions using multiple auxiliary modules and adapts the entire planning process dynamically.

Based on memory and LLMs as the core of planning, experimental results demonstrate that the CPMI exhibits the best few-shot performance in the current ALFRED environment without any fine-tuning. Our framework is highly extensible and fully demonstrates that in today’s rapid development of an LLM, the embodied intelligence task can be transformed into a downstream task of an LLM, which also provides a new direction for future work. For example, with the increased capabilities of LLMs or the development of low-level controllers, it is possible to indirectly influence the ability of CPMI to be used conveniently, whether for domestic or industrial robots.

The ultimate objective of artificial intelligence should be an agent that possesses human-like abilities. Therefore, we tend to align it with the essential behavioral patterns of human beings, where the brain is used to remember and think. In contrast, the eyes are used to extract visual information. We believe that as the trend for developing LLMs continues, the embodied task will become more comprehensive in terms of mapping AI to the real world. The primary constraint of our present design is that the environment in the real world is not static (e.g., human intervention), leading to differences between our memories and the real environment; how to dynamically memorize, acquire perceptions, and abstract more high-level memories is a direction to be explored in the future.

## Conclusion

We propose the CPMI, a lightweight embodied task framework with strong extensibility and robustness that generates sequences of tasks under corresponding instructions through an LLM. In CPMI, we center the task planning around a Memory Module, which allows the agent to have the ability to memorize as well as abstract experience. Finally, it shows through evaluation on ALFRED that CPMI achieves SOTA performance under the few-shot and increases performance under successive instructions as the experience is accumulated.

However, research into advanced memory abstraction is currently understudied. Our goal is to extract more relevant information during agent execution, and in the future, we plan to refine our abstract process and apply it to more realistic scenarios.

## Data Availability

Data will be available upon request.
